# Targeted dose delivery of *Mycobacterium tuberculosis* in mice using silicon antifoaming agent via aerosol exposure system

**DOI:** 10.1371/journal.pone.0276130

**Published:** 2022-10-13

**Authors:** Uma Shankar Gautam, Rosemarie Asrican, Gregory D. Sempowski

**Affiliations:** 1 Duke Human Vaccine Institute, Duke University School of Medicine, Durham, North Carolina, United States of America; 2 Departments of Medicine and Pathology, Duke University School of Medicine, Durham, North Carolina, United States of America; Translational Health Science and Technology Institute, INDIA

## Abstract

*Mycobacterium tuberculosis* (Mtb) is an intracellular pathogen that forms aggregates (clumps) on solid agar plates and in liquid media. Detergents such as Tween 80/Tyloxapol are considered the gold standard to disrupt clump formation in Mtb cultures. The presence of detergent, however, may generate foam and hinder Mtb aerosolization thus requiring addition of an antifoam agent for optimal Mtb aerosol-based procedures. Aerosol inhalation can be technically challenging, in particular to achieve a reproducible inhaled target dose. In this study, the impact of an antifoam, the silicon antifoaming agent (SAF), on Mtb aerosolization and whole-body mouse aerosol infection was investigated. A comparative study using SAF in a liquid suspension containing *Mycobacterium bovis* BCG (*M*. *bovis* BCG) or Mtb H37Rv did not cause any adverse effect on bacterial viability. Incorporation of SAF during mycobacteria inhalation procedures revealed that aerosolized mycobacterial strains were maintained under controlled environmental conditions such as humidity, temperature, pressure, and airflow inside the aerosol chamber. In addition, environmental factors and spray factors were not affected by the presence of SAF in mycobacterial cultures during aerosolization. Spray factor was significantly less during aerosol procedures with a low-input dose of mycobacteria in comparison to high-dose, as predicted. The mycobacterial load recovered in the biosampler (AGI) was ~2–3 logs lower than nebulizer or input bacterial load. A consistent Mtb bacillary load determined in mouse lungs indicates that SAF does not affect mycobacteria aerosolization during the aerosol generation process. These data confirmed that 1) SAF prevents formation of excessive foam during aerosolization, 2) SAF had no negative impact on mycobacterial viability within aerosol droplets, 3) Mtb droplets within aerosol-generated particles are well within the range required for reaching and depositing deep into lung tissue, and 4) SAF had no negative impact on achieving a target dose in mice exposed to Mtb aerosol.

## Introduction

*Mycobacterium tuberculosis* (Mtb) is an intracellular pathogen that is transmitted by aerosol from infected individuals. Mtb aerosol generated by coughing or close contact with a person who has active tuberculosis (TB) not only increases the risk of disease transmission but has been attributed to several outbreak reports [1–5]. Mtb aerosol production and distribution in TB disease has not been broadly studied and requires detailed investigation [6]. Interventions targeting aerosol transmission and inhaled therapies to TB could be effective in lowering TB transmission [7]. Mtb forms clumps that are shown to be associated with severe illnesses in humans and bacilli forming large aggregates kill the majority of macrophages upon infection [8, 9]. Mtb also forms aggregates in cultures grown *in vitro*. These clumps lead to inaccurate bacterial counts and dilution factors in liquid cultures. Therefore, it is necessary to disrupt the bacterial clumps. Mtb clumps in liquid cultures can be disrupted by adding detergents such as Tween 80 [10–13] or Tyloxapol [14, 15]. However, liquid suspension of Mtb containing detergent generates foam that may limit aerosolization and dynamics of aerosol particles during aerosol generation procedures and makes the aerosol inhalation technically challenging, in particular achieving a reproducible inhaled target dose. Several laboratories have reported the use of detergent and silicon based antifoam during aerosol generation procedures [14–19]. Others have reported that silicon based antifoam does not affect cell viability but increases yield of recombinant protein in cell culture [20, 21]. Addition of detergent is required to dissociate clumps whereas addition of antifoam inhibits foam formation, as well as ensures that the bacterial suspension does not undergo unnecessary changes while retaining the test sample in stable form [22] during aerosol procedure.

One of the characteristics of an antifoam agent is its ability to resist any physiochemical changes that could compromise its antifoam activity overtime. Therefore, antifoam activity of a chemical agent may have a role in the regulation of bio-suspension in its native form. A number of antifoams have been used to inhibit foam formation during Mtb aerosol generation and delivery [14–17, 22]. Over time however, some of these have been discontinued or are no longer available. Thus, there is a need for new antifoam agents to be evaluated and optimized for their solubility, non-toxic nature, effectiveness in suppressing the foam formation and redundancy in anti-bacterial affect and maintaining Mtb viability. Aerosol procedures are conducted using liquid suspension in small-volume nebulizers [14]. The AeroMP aerosol generation system delivers compressed gas through a jet, causing a negative pressure in the aerosol machinery [14, 15]. The Aerosol machinery maintains controlled environmental settings inside the aerosol chamber with compressed air and converts bacterial suspensions into aerosolized droplets that are deposited into the target animal’s lower respiratory tract [14, 17].

Mtb infection using aerosol inhalation procedures offers an unique opportunity to establish a target dose of pathogen at the site of infection. Aiming for a targeted bacterial dose is critical to understanding the establishment of Mtb infection and progression to active disease. It is important to ensure appropriate delivery of aerosolized particles for optimal outcomes in aerosol inhalation procedures. It has been reported that viability of mycobacteria declines in aerosol droplets, as well as in the nebulizer during the process of aerosolization [23, 24].

The current study assessed the ability of silicon antifoaming agent (SAF), a water-based silicon emulsion, to control foam during aerosol inhalation exposures. Specifically, the studies investigated SAF as a foam inhibitor in a controlled aerosol chamber mimicking naturally occurring environmental conditions. Studies were also performed to determine whether SAF continues to control foam or reduce preexisting foam in suspension. This approach resembles the prior studies in the field as the employed aerosol exposure system uses universal and controlled conditions as previously described [11, 13, 16, 17, 25]. Furthermore, this study was conducted to test the antifoam activity of SAF under ordinary salt conditions and physiological pH (0.155 M NaCl, pH 7.4), and in the absence of any chemical agent that could impact the activity of SAF in the bacterial suspension.

Overall, the studies performed demonstrate that SAF can control foam in bacterial suspension of various mycobacterial species during aerosol generation. This study summarizes the function, importance, and use of antifoam in Mtb cultures during aerosol procedures. This study also provides the ability to predict the target dose of Mtb aerosol infection using SAF via inhalation exposure system.

## Materials and methods

### Silicon antifoaming agent

Silicon antifoaming agent (Cat# 1077430100) was purchased from MilliporeSigma. Silicon antifoaming agent (SAF) was prepared fresh (200 ppm) in PBSTy solution that contains a sterile mixture of 1x PBS, pH 7.4 (Cat# 70011, Invitrogen) and 0.05% Tyloxapol (Cat# T8761, MilliporeSigma). The PBSTy containing SAF was mixed by vortexing and pipetting several times (a vigorous stirring may be necessary to homogenize the emulsion), followed by filter sterilization (Cat# SCGP00525 0.22 μm, Millipore) prior to use.

### Foam measurement

Foam formation or suppression was measured in tubes containing PBSTy without and with antifoam SAF by vigorous shaking and end-over mixing during measurement up to 20 minutes (the maximum time bacterial cultures remain in nebulizer (NEB) and biosampler (All Glass Impinger; AGI) during aerosol procedure. The foam volume (corresponds to foam height) was measured using a ruler in all tubes immediately after vigorous shaking (0 min) and then at 5 min interval for a total of 20 min. The foam volume that retained in the tubes containing PBST (PBS + Tween) with and without SAF at 5 min, 10 min, 20 min time interval with respect to ‘0 min’ were plotted as fold decrease in foam formation.

### Mycobacteria and growth

Frozen stocks of *Mycobacterium tuberculosis* (Mtb) strain H37Rv (a kind gift from Claire Smith, Assistant Professor, Duke University School of Medicine) and *Mycobacterium bovis* (*M*. *bovis*) strain BCG (Cat# 35733, *Mycobacterium bovis* Karlson and Lessel ATCC 35733 TMC 1010 BCG Danish) were cultured in Middlebrooks’s 7H9 media (Cat#271310, BD) supplemented with 10% OADC (Cat#212351, BD), 0.05% Tyloxapol (T8761, Sigma-Millipore), 0.5% Glycerol (G9022, Sigma-Millipore) and referred to as 7H9 complete media. The 7H9 complete media was filter sterilized (0.22 μm, Cat#430769, Costar) prior to use. Mtb was cultured to logarithmic phase (OD_595_ ∼0.3–0.4) in a vented flask (Cat# CLS431401, Sigma-Millipore) by shaking at 220 rpm and 37°C. The freshly grown cultures were serially diluted and plated on Middlebrook’s 7H10 agar complete media to determine viable CFU counts. Colony forming units (CFUs) were enumerated 21 days after incubating agar plates at 37°C [26]. The target OD_595_ required as input to achieve a desired experimental dose for aerosol infection was determined in this manner. For mycobacteria viability testing, Mtb H37Rv and *M*. *bovis* BCG were incubated without SAF with SAF (cultures that remain in contact with SAF for a short (20min) and longer (24hr) duration) and bacilli counts were determined for each time point by CFU assay. All experimental work with risk group 3 pathogenic Mtb strain was conducted in a biosafety level-3 (BSL3) facility [Duke Regional Biocontainment Laboratory (RBL), Durham, NC].

### Mice

Animals were acclimatized in animal holding area for one week prior to aerosol exposure. The vivarium rooms are environmentally controlled at a 21°C and 50% relative humidity. C3HeB/FeJ male mice (6 to 8-week-old; Jackson Laboratory, Bar Harbor, ME) were infected with ~300 CFUs of bacilli via aerosol (Mtb infected group) or did not receive SAF or Mtb (control group). An initial bacterial deposition was determined in Mtb infected group by CFU assay of lung homogenates day 1 post infection, as previously described [14]. The remaining mice in each group were supervised for their health and their body weight and temperature were also measured daily for up to 6 weeks. Clinical measurements for Mtb-infected versus control mice were compared at completion of the study. Mice were euthanized via CO_2_ asphyxiation and cervical dislocation prior to collecting mouse organs (lung and liver). Bacterial burdens were also determined in the lung and liver of Mtb infected mice by CFU counts at week 6 post infection.

Duke animal facilities are accredited by the American Association for Accreditation of Laboratory Animal Care and licensed by the U.S. Department of Agriculture. All relevant procedures were approved (study protocol approval number A081-20-04) by the Duke Institutional Animal Care and Use Committee (IACUC) and the Duke Institutional Biosafety Review Committee. All animals were routinely cared for as per the guidelines prescribed by the National Institutes of Health Guide to Laboratory Animal Care. Humane endpoints were predefined in this protocol. The animals were group-housed in appropriate social settings in accordance with the guidelines of AAALAC, which annually inspects all facilities. The IACUC performs semiannual and annual inspections of the Duke ABSL2/3 facility to certify compliance with highest possible levels of housing conditions, feeding regimens, and environmental enrichment.

### Aerosol generator assembly

The aerosol generator assembly (Biaera Technologies, LLC, USA) in the Duke RBL was used for all aerosol experiments. A detailed diagram of aerosol chamber exposure system that includes components such as biosampler, nebulizer, chamber pressure sensor, relative humidity, temperature controller, sampling port and connection of the port to animal exposure chamber etc., and their description has been previously published [14]. The Duke RBL is a fully commissioned BSL3/ABSL3 facility housed in the Global Health Research Building (GHRB), Duke Human Vaccine Institute (DHVI), Duke University School of Medicine, Durham, NC. Standard operating procedures were followed to maintain sterile conditions and whole-body aerosol exposures carried out as previously described [14, 15]. The biosampler, nebulizer, 6-jet nozzles, and O-rings were routinely examined for any deposition or damage such as fraying or decay. These components were properly cleaned using Sparkleen (Cat#04-320-4, Fisher Scientific) and warm water in a Precision Needle-Tip Squeeze Bottle (Cat#1902T61, McMaster-Carr) followed by thorough rinsing or were replaced when required. All components were routinely maintained as per manufacturer’s instructions (Biaera Technologies).

### Aerosol procedure

The aerosol exposures were conducted with AeroMP system [14, 27]. For aerosol procedures, freshly grown bacterial cultures were used without sonication or syringe passaging. Target optical density (OD_595_) required to achieve a desired experimental Mtb dose in aerosol procedure was first optimized by doing a “mock” or sham experiment that used BCG but no animals. The bacterial suspension of Mtb or *M*. *bovis* was always prepared in PBSTy containing SAF and used for aerosol procedures conducted, unless otherwise mentioned. Ten-fold serial dilutions of bacterial cultures (input) and those recovered after aerosolization (remaining contents from NEB and AGI) were immediately plated on 7H10 agar plates to determine viable CFU counts. Mouse whole body aerosol infections were carried out following standard operating procedures as described previously [14]. Briefly, 20 minutes each for exposure and purge cycles were allowed during animal infection. After the chamber purge cycle was complete, animals were allowed to sit for an additional 20 minutes and then removed from the exposure chamber (Madison) and transferred to biocontainment cages in the Duke RBL ABSL3 containment facility.

## Results

### Silicon antifoam effectively suppresses foam formation and does not have an adverse effect on mycobacteria viability

**Fig 1:**
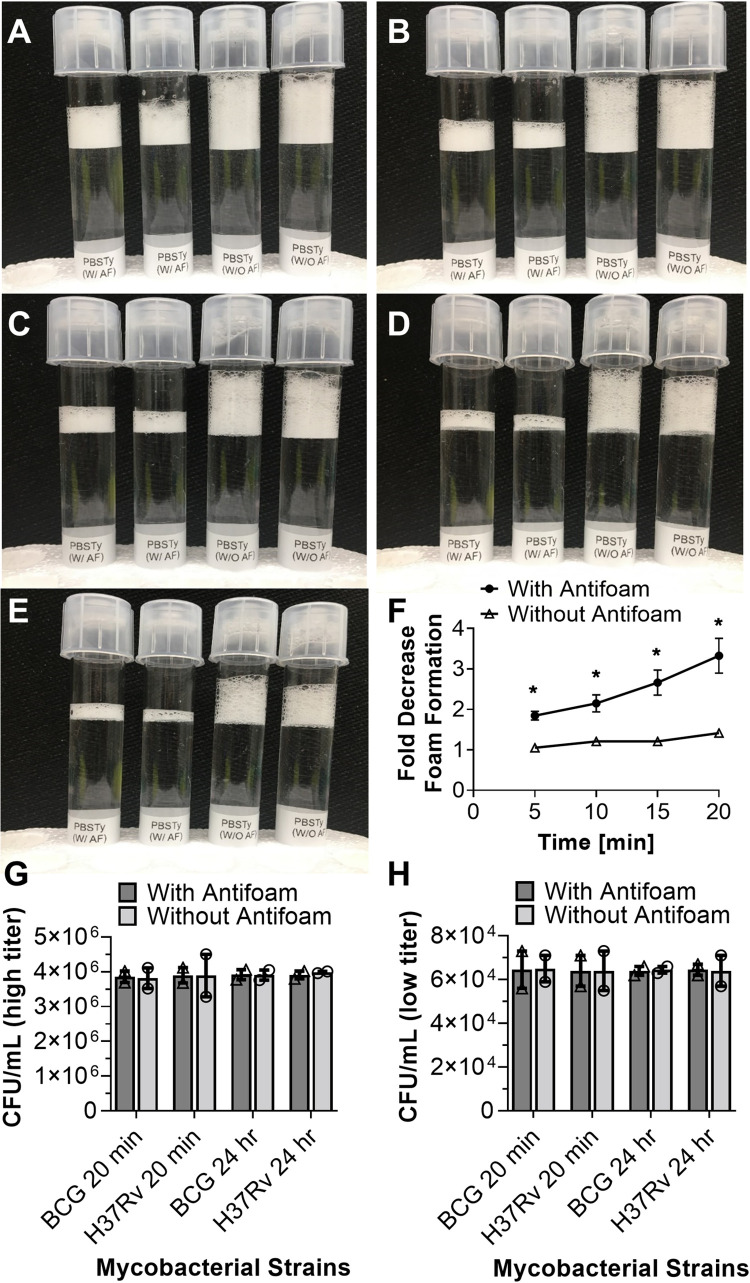
Testing SAF and foam formation. Tubes containing PBSTy showing foam suppressed (with SAF) or retained (without SAF) at 5 minute interval for the duration of 0–20 min (panels A-F). Graph in panel ‘F’ extrapolated from panels ‘A-E’. Viability testing of *M*. *bovis* BCG and Mtb H37Rv by CFU assay without SAF and with SAF in contact with mycobacteria for 20 min and 24 hr (day 1) for two CFU titers tested; ~4e6 (G), ~6e4 (H).

An initial experiment was conducted to test whether addition of SAF reduces foam in a detergent-suspension of PBS (PBSTy). The tubes with and without SAF were examined simultaneously to measure the amount of foam that was inhibited or sustained over time (Fig 1). A vigorous shaking of tubes with SAF notably produced less foam and effectively reduced foam during 20 minute study duration (Fig 1). These results clearly indicate that SAF is a foam inhibitor that not only retains its antifoam activity, but also controls the rate of foam formation during mixing (Fig 1A) and over time (Fig 1A–1E). In comparison, tubes containing PBSTy only (without SAF) had foam present that did not reduce over time (Fig 1A–1E). These results demonstrate that SAF brings down foam (> 3-fold, Fig 1F) in liquid suspension.

Several findings have reported successful use of silicon based antifoam (600 ppm) in aerosol procedures [18] or 1000 ppm in cell based assays [28] which is 3–5 fold hither than SAF amount used in this study. However, it is highly likely that exposure to new antifoam SAF may contribute to the sensitivity and viability of aerosolized mycobacteria therefore an in-vitro experiment was conducted to check mycobacteria viability for an extended period of time (up to 24 hr) than the standard aerosol exposure limit (20 min). Mycobacteria viability was examined by first incubating Mtb H37Rv and *M*. *bovis* BCG with SAF (test group) and without SAF (control reference group). Two groups of mycobacterial preparations containing high titer (~4e6 CFU counts) and low titer (~6e4 CFU counts) of Mtb H37Rv or *M*. *bovis* BCG were incubated with and without SAF in PBST suspension by mixing on a rocker for 20 minutes (equivalent to standard aerosol exposure time) as well as for a longer period (24hr) than standard exposure time limit. Mycobacterial cultures were serially diluted and plated to determine viable CFU counts in both groups from each time point. Comparable CFU counts were obtained for both Mtb H37Rv and *M*. *bovis* BCG in experimental tubes with SAF, as well as control tubes without SAF (Fig 1G and 1H). These results show that addition of SAF had no negative impact on Mtb H37Rv as well as *M*. *bovis* BCG viability tested for high- (~4e6 bacilli, Fig 1G) and low-mycobacterial-titer (~6e4 bacilli, Fig 1H) for short (20 min) or longer (24 hr) contact time with SAF.

### Silicon antifoaming agent does not affect chamber environmental conditions or jet nebulizer performance during aerosol generation

**Fig 2:**
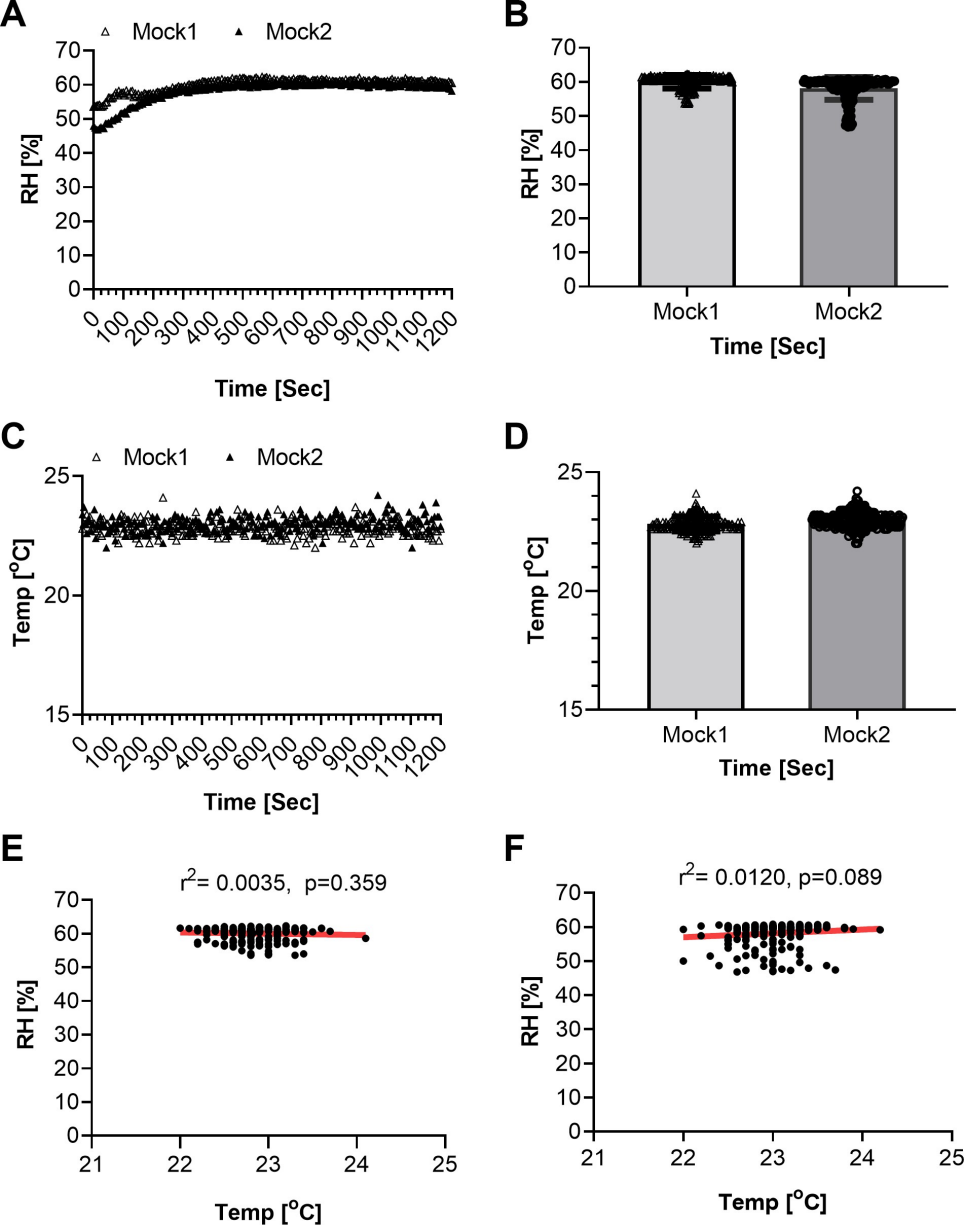
Environmental factors measured in chamber during mock aerosol experiment. Environmental factors measured for mock1 and mock2 during aerosol procedures by AeroMP system; range of relative humidity plotted with respect to time (0–1200 sec) (A), mean relative humidity (RH) mock1 (60.05%), mock2 (58.12%) plotted versus time (0–1200 sec) (B), temperature recorded for mock1 and mock 2 every 5 sec up to 1200 sec (20 min) and plotted with respect to time (C), mean temperature recorded for mock1 (22.8°C), mock2 (22.9°C) plotted versus time up to 20 min (0–1200 sec) (D), range of relative humidity plotted versus temperature for mock1 and their linear regression (r^2^ = 0.0035, P = 0.359) (E), relative humidity plotted versus temperature measured for mock2 and their linear regression (r^2^ = 0.012, P = 0.089) (F).

Environmental conditions inside the aerosol chamber must be maintained throughout aerosol generation procedures, as they are key factors in determining aerosol particle size and nebulizer performance. In particular, water evaporation during aerosol procedures can reduce the temperature of an aerosol, which may result in an increase in viscosity of bacterial suspension and a decrease in nebulizer output. The AeroMP system monitors humidity and temperature, and simultaneously records this information. These parameters were examined after each mock aerosol procedure. Relative humidity (Fig 2A and 2B) and temperature (Fig 2C and 2D) started at a comparable level for both mock (1 and 2) doses tested during aerosolization. However, a slight delay in achieving appropriate humidity level was noted for mock1 in comparison to mock2 at the beginning of the run (Fig 2A). On average, relative humidity was recorded in the range of 58–60% and the temperature was ~23°C during mock1 and mock2 aerosolization [20 minutes (1200 seconds) max duration] (Fig 2). These results indicate that relative humidity and temperature were maintained inside the chamber throughout the aerosol procedure. To predict any relationship between humidity and temperatures, a linear regression analysis was conducted. The analysis revealed these two variables are independent and their relative values cannot predicate any relevant correlates either for mock1 (Fig 2E, r^2^ = 0.0035) or mock2 (Fig 2F, r^2^ = 0.0120). The power of regression (r^2^) was weaker for mock1 than mock2.

### A predictive target dose can be achieved based on input bacterial dose present

**Fig 3:**
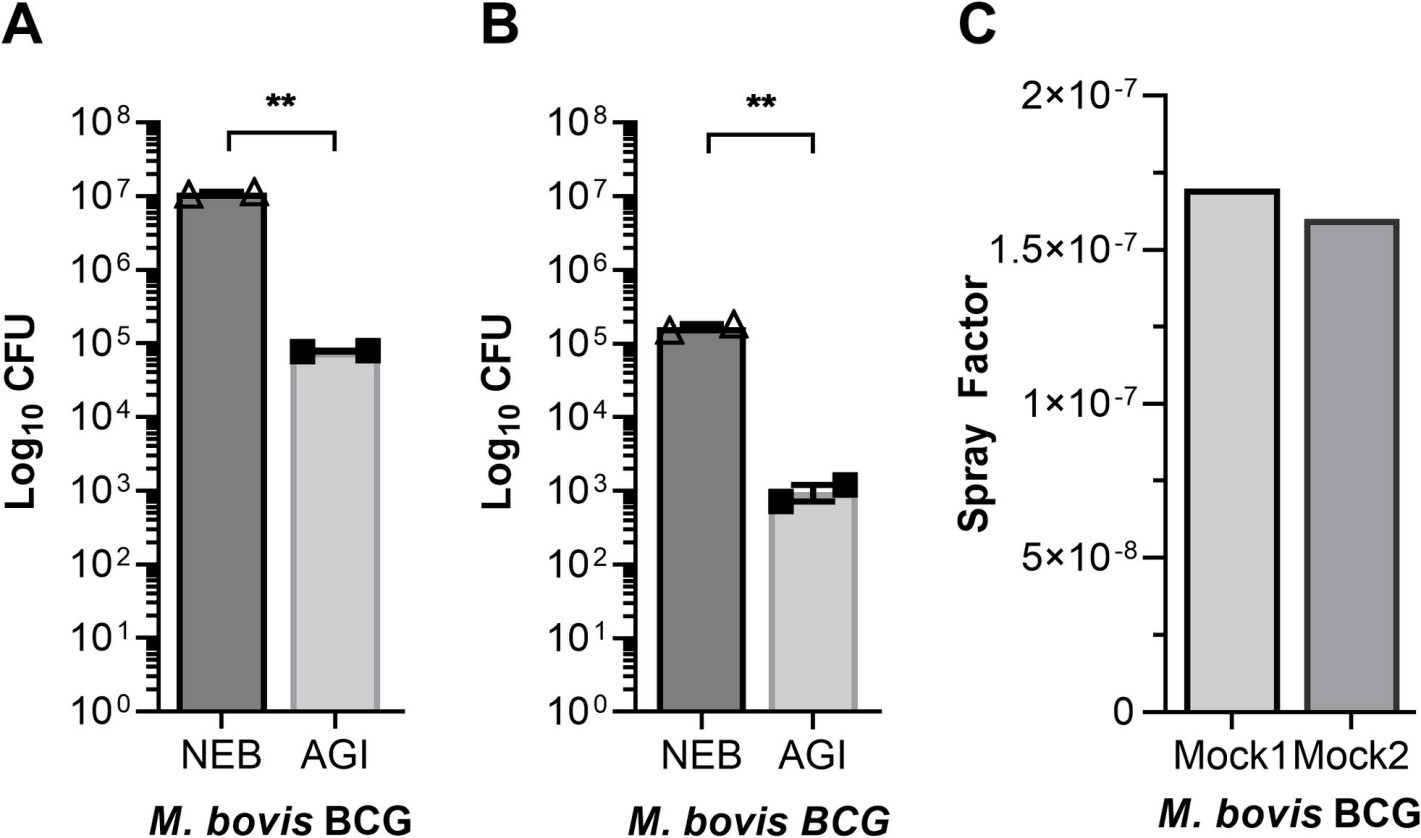
Bacterial burden in aerosol assembly during mock experiment. Bacterial burdens obtained in nebulizer (NEB) and biosampler (AGI) plotted as total CFUs; mock1 (A), mock2 (B). Spray factor for each mock1 and mock2 aerosol procedures (C). Data are mean ± SEM, unpaired t-Test, **P = 0.0016 for mock1, **P = 0.0090 for mock2 using Graphpad prism 9.

*Mycobacterium bovis* BCG (*M*. *bovis* BCG) was used in mock experiments (no mice were used during mock1 and mock2 aerosol runs) since it does not require a specialized high-containment laboratory. Mock aerosol experiments were conducted using different doses of *M*. *bovis* BCG; mock1 (1.1e7), mock2 (1.7e5) that brings ~100-fold difference between two input doses (Fig 3). Mock aerosol generation experiments were conducted with *M*. *bovis* BCG containing SAF to confirm if a definite target dose can be achieved as a correlate of input bacilli present prior to aerosol procedure. A significant component of this objective was to determine if there are differences in numbers of bio-aerosol particles generated in the nebulizer and collected in the AGI from bacterial suspension with high- (~10^7^ CFUs) versus low-titer (~10^5^ CFUs) preparations. A drop (~2 log) in bacilli counts from NEB to AGI was noted during aerosol procedure conducted using mock1 and mock2 (Fig 3A and 3B). These enumerations are based on CFU data normalized based on total volume present at the beginning of each run and the volume remaining (~56–63% loss in volume) in the nebulizer and AGI upon aerosol procedure termination. These measurements are in agreement with previous reports where authors reported a similar loss in volume during aerosol runs [10, 17, 25, 29]. The viable bacilli that remain in NEB and AGI help determine CFU counts that should be provided as input in the nebulizer to achieve a definite target dose in mice challenged with Mtb.

### Spray factor depends on initial bacterial load present in inoculum

Spray factor was evaluated to determine if mixing of SAF with mycobacteria prior to aerosol exposure causes any loss of bacterial agents. Spray factor was calculated for mock1 and mock2 aerosol runs following the method previously described [17]. Spray factor, a ratio of mycobacteria aerosol concentration in biosampler (viable CFU counts, AGI) to nebulizer (viable CFU counts, NEB) is used to assess aerosol generation performance. Aerosolization of *M*. *bovis* BCG revealed spray factor as a correlate of bacterial input dose as expected. The spray factor with low bacteria input was relatively lower than higher input dose (Fig 3C). These results demonstrate that presence of SAF during aerosol procedure had no negative impact in achieving a target mycobacterial dose. Next, the standardized conditions as above were adopted to evaluate SAF in conducting aerosol procedure to challenge mice with virulent Mtb strain H37Rv to achieve a desired bacterial dose and to assess any impact of SAF on environmental factors within aerosol chamber.

### Determination of environmental factors and spray factor in the aerosol chamber during Mtb-aerosolized mice infection

**Fig 4:**
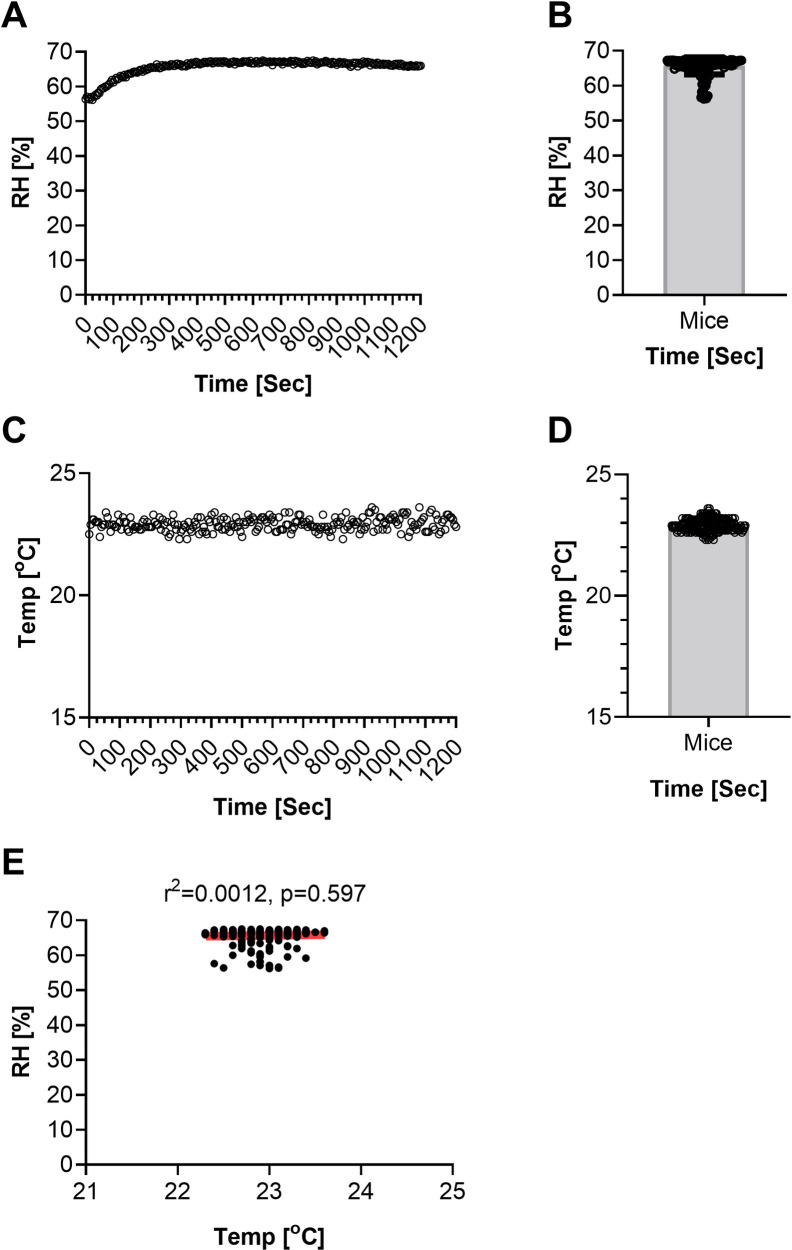
Environmental factors measured in chamber during Mtb-aerosol exposure of mice. Range of environmental factors measured during mouse aerosol procedures using the AeroMP system; range of relative humidity plotted with respect to time (0–1200 sec) (A), mean relative humidity plotted versus time (B), range of relative humidity plotted versus the temperature measured over time (0–1200 sec) (C), range of temperature recorded every 5 sec plotted with respect to time (D), mean temperatures plotted versus time (0–1200 sec) and their linear regression (r^2^ = 0.0012, P = 0.597) (E).

**Fig 5:**
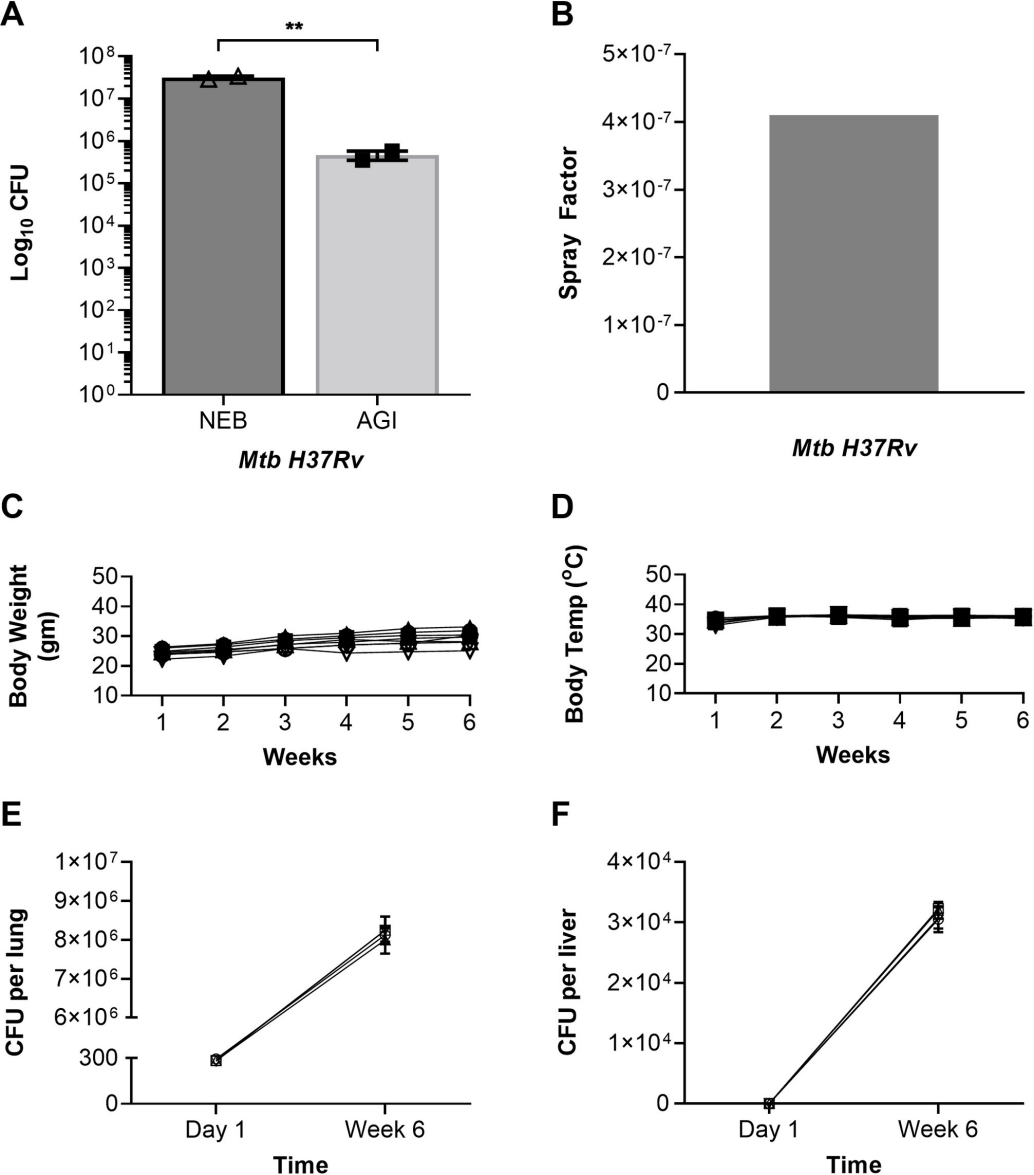
Bacterial burden in aerosol assembly during Mtb-aerosol exposure of mice and post Mtb-infection. Bacterial burdens obtained in nebulizer (NEB) and biosampler (AGI) plotted as total CFUs (A), spray factor measured for Mtb-aerosol exposure of mice (B), average body weight (C) and temperature (D) of mice measured up to 6 weeks; closed shapes (Mtb-infected), open shapes (control mice that did not receive Mtb). Bacterial burdens day-1 and week-6 post infection of mice; lung (E), liver (F). CFU data are from duplicate plating; results in panels E and F are expressed as CFUs in the entire tissue; data are means ± SEM. For panel A, ** P = 0.0069, unpaired t-Test using GraphPad prism 9.

Environmental factors such as humidity and temperature were also recorded inside the chamber during Mtb H37Rv-aerosol exposure of mice. These parameters were examined at completion of the aerosol procedure. The relative humidity was slightly lower (~57.4%) at the beginning (during first 60 seconds of the run) but increased to 62.4% within the next 60 seconds of the exposure cycle, with a further increase by 1% in 240 seconds (Fig 4A and 4B). The relative humidity levels became constant thereafter during the remainder of exposure cycle (1200 sec max duration). Overall, relative humidity was recorded in the range 57.4–65% (Fig 4A and 4B) and the temperature at ~23°C (Fig 4C and 4D) during the entire aerosol procedure conducted with mice present. On average, relative humidity was recorded as 65.6% and the temperature at ~23°C during Mtb H37Rv-aerosolized mice infection (Fig 4A–4D). A 2–7% difference was noted in the humidity levels recorded for aerosol procedures conducted without mice (Fig 2A and 2B), in comparison to aerosol procedures conducted when mice were present (Fig 4A and 4B). These data confirm that temperature was maintained at ~23°C inside the chamber throughout the aerosolized Mtb H37Rv exposure, despite a slight variation in relative humidity recorded for mock1 in comparison to mock2 (Fig 4A). A similar trend of increase in humidity rate was recorded during mouse aerosol procedure (Fig 2A). Average humidity recorded was slightly higher for mock1 (humidity 60%, input bacterial dose 8.3E6) than mock2 (humidity 58%, input bacterial dose 1.3E5). The humidity levels recorded for mock1 and mock2 however were not statistically different (Fig 2B). A slightly higher relative humidity (65.4%) was achieved during Mtb aerosol exposure of mice (Fig 4A and 4B) than mock experiments (relative humidity 58–60%, Fig 2A and 2B). Linear regression analysis revealed these two variables, humidity and temperature, are independent and their relative values cannot predicate any relevant correlates (Fig 4E, r^2^ = 0.0012) during aerosol run. Spray factor was determined during aerosol procedures conducted with and without mice present. In comparison to mock aerosol experiments (with *M*. *bovis* BCG contact but no mice present, Fig 3), Mtb H37Rv-aerosolized infection of mice resulted in a higher spray factor (~2.5 fold) due to ~4-fold higher Mtb H37Rv bacilli used as input (Fig 5A, input bacteria 2.4E7) than mock1 (Fig 3A, input bacterial dose 8.3E6) or mock2 (input bacterial dose 1.3E5). The spray factor data obtained for mock (Fig 3C) or mice (Fig 5B) indicate that SAF has no negative impact on Mtb aerosol delivery and achieving a desired Mtb dose in mice.

### SAF has no adverse effect on aerosolized mycobacteria and mice

Mice challenged with aerosolized Mtb H37Rv suspension containing SAF (Mtb-infected) or mice that did not receive any SAF or mycobacteria (control) were carefully monitored for any notable symptoms thereafter. Mtb-infected mice (~300 CFU/mouse) were measured for (a) bacterial burdens in lung at day 1 post infection (b) bacterial burdens in lung and liver at week 6 post infection, (c) daily measurement of mouse body weight and temperature of mice in all groups up to 6 weeks. On day 1 post infection or longer (up to 6 weeks) mice did not develop any adverse signs or symptoms, and had no weight loss (Fig 5C) or fever (Fig 5D). No differences in mouse body weight or temperature measures were observed in Mtb-infected versus the control mice that did not receive any SAF or Mtb. A comparable body weight of Mtb infected mice group (average weight at week 6 = 28.7 gm) and control group (average weight at week 6 = 26.6 gm) (Fig 5C) and temperature of Mtb infected mice group (average temperature at week 6 = 35.8°C) and control group (average temperature at week 6 = 35.4°C) (Fig 5D) recorded are indicative as a safety measure of SAF usage during Mtb aerosolization. Overall, mice were infected with a desired target dose of Mtb (averaged ~300 bacilli implanted in mouse lung on day 1 post infection) and bacterial load increased to approximately 8.2 x10^6^ in lung (Fig 5E) and 3.14 x10^4^ in liver (Fig 5F) by week 6. No bacilli were detected in liver on day 1 post infection as expected (Fig 5F). The aerosol transmission strategies were fully effective with multiple doses of bacterial strains; low versus high or mock versus Mtb aerosolized mouse infection, leading to a conclusion that Mtb aerosol efficacy proved successful aerosol transmission during aerosol run. Results of mouse infection clearly demonstrate bacilli deposition at the target site and multiplication in the lung (Fig 5E), as well as their dissemination to liver during week 6 post infection (Fig 5F). This implies that bacilli exposed to SAF not only internalized and implant successfully in mouse lung but are capable to multiply and disseminate during the course of infection. The extrapulmonary infection determines that bacilli remain infectious in mice. These results determined that SAF does not impact Mtb infectivity.

## Discussion

An assembly used to generate Mtb aerosol for animal inhalation studies is a closed system that is controlled by compressed air to generate a negative pressure and thereby produce aerosol particles of a definite size [10, 11, 16, 17, 30]. Compressed air should be consistent for successful aerosol formation by the AeroMP or similar systems [11, 14, 15, 17, 26, 31]. The settings for a closed aerosol assembly should not modulate any of the environmental factors during aerosol generation [14, 15, 17]. The aerosol particles are produced in a nebulizer [14, 15], passed on to a biosampler and inhaled by the target animals, resulting in inhalation of the aerosol droplets and dissemination in the animal [11, 12, 16, 32]. Various aerosol assemblies exist that have successfully utilized nebulizers and are applicable for respiratory infectious disease research [16, 17, 30].

Saini and others reported adding antifoam Y-30 emulsion for a successful Mtb aerosol generation [15, 25]. Redman et al. used antifoam Y-30 and have shown that Mtb aerosol produced in jet nebulizers has uniform particle size [25]. Additionally, antifoam Y-30 [22] and antifoam 289 [33] have been shown to increase protein production yield since they prevent excessive foam formation in shaking cultures. The antifoam Y-30 is an aqueous silicon emulsion that has been discontinued by Sigma-Aldrich, so there is a need to identify and test new antifoam agents. Both, antifoaming agent Y-30 (Ref# A5758) and silicon antifoaming agent (Ref# 1077430100) are silicon based aqueous antifoaming agents, but the exact details of their composition are considered proprietary information by the manufacturer. The antifoaming agent Y-30 (Ref# A5758) was a product from Sigma-Aldrich, whereas Silicon antifoaming agent is a product which comes from the EMDMillipore. Antifoaming agent Y-30 (Ref# A5758) was the only product of the two with a disclosed concentration (30% aqueous emulsion), likely because the concentration of antifoam agent in silicon antifoaming agent (Ref# 1077430100) is adjusted to meet the density specification 0.98–1.01 g/mL listed by the manufacturer.

One of the characteristics of an antifoam agent is its ability to resist the physiochemical changes to keep a solvent in its native form. The antifoam in general works by rupturing the foam films that cause destabilization of foam by repulsion or a similar mechanism of action with liquid drainage followed by bridging of the liquid film by silica particle present in antifoaming agent that results in foam rupturing [34, 35]. Others have reported bridging-stretching mechanism of action that allows a biconcave bridge formation by silica particle present in antifoaming agent with the foam followed by stretching of the bridge to ruptures the film and destabilizes foam [36, 37]. In the context of antifoam action, Kulkarni et al. [38] have reported an antifoam mechanism in which silicon based antifoam droplets approach the liquid-air contact at foam surface and control the foam because of their low surface tension properties. These authors explained that antifoam influences the defoaming process through charge repulsion mechanism between the interfaces of water and antifoam. In this study, silicon antifoaming agent was examined to determine if it could be safely employed and optimized to use in Mtb aerosol inhalation procedures in mice.

Aerosol generation using liquid suspension is a complex procedure since its operation is time- and solubility-dependent. The entire aerosol procedure takes approximately 1 hour to complete, of which the first 20 minutes are for Mtb aerosolization [10, 14, 15, 17, 26]. Mtb forms aggregates in liquid cultures [8, 39–42] that are disrupted by adding detergents such as Tween 80 or Tyloxapol in small quantities (0.05–0.1%) [10, 14, 15, 26, 43]. Antifoam is added to Mtb cultures that destabilize foam during aerosol procedures. Therefore, antifoam activity has been correlated with antifoam efficacy during aerosolization procedures [10, 14, 16, 17, 30]. Silicon based antifoams Antifoam does not induce any variability or affect the viability of bacilli besides controlling foam during aerosol procedures [14, 16, 17, 30]. Similarly, others have also reported that silicon based antifoam does not affect bacterial cell viability [20]. These authors compared multiple silicon based antifoams and found that silicon based antifoam not only reduced foam formation in bacterial cultures but increased protein production. The results present in this study have no adverse effects of SAF on mycobacteria viability (Fig 1G and 1H) or mice (Fig 5).

The findings from this study show that silicon antifoam is safe and that it not only leads to better control of foam in Mtb cultures, but it substantially reduces the rate of foam formation (Fig 1). This was associated with a target dose of ~300 Mtb bacilli delivered to mouse lung via aerosol (Fig 5). These data demonstrate an establishment of low-dose aerosol infection in mice using Mtb H37Rv cultures containing SAF (Fig 5). This is in agreement with the observation that spray factor was improved (higher yield) (Figs 3C and 5B) when higher Mycobacterial dose was used as input (Figs 3A and 5A) in aerosol procedures Thus, Mtb preparations containing SAF produce aerosol particles and their viability was not affected by the inclusion of silicon antifoam agent (Figs 1, 3 and 5). These results have implications in that SAF can serve as a substitute for other antifoam agents used in aerosol procedures. The current availability and use of SAF as an antifoam appears to overcome any limitation or non-availability of a particular antifoam. The results from this study suggest that SAF prevents foam formation in Mtb suspension while being in contact with a pathogen for short (20 min) versus longer duration (24 hr) without affecting its viability (Fig 1G and 1H).

Analysis of aerosol machinery emissions (AGI) during mice infection or mock experiments has revealed the presence of mycobacteria from all aerosol runs conducted with a variety of bacterial input doses (Figs 3 and 5). All aerosol procedures conducted confirm the presence of mycobacteria in the remaining liquid contents of aerosolization (NEB/AGI) (Fig 3) as well as in mouse lungs (Fig 5). Results of mice challenged with Mtb concluded that aerosols transmission was successful (Fig 5E and 5F). The factors affecting airborne transmission and bacterial load in different-dose not only confirm the stability of Mtb in aerosols but the dose-response relationship for each *Mycobacterium* strain tested (the probability of infection for a given exposure with a definite number of bacilli). These results emphasize that Mtb strain used for mouse infection had the potential to initiate infection since they remain infectious in contact with SAF (Figs 1G, 1H and 5E).

Whereas SAF is an important antifoam (Fig 1) and its activity correlates with decreasing foam in liquid suspension, its solubility is a bit compromised due to its sticky nature. The approaches targeting increasing the solubility of SAF in solution may be attempted in future studies. Moreover, other antifoams may also be evaluated to test if they are more effective than SAF at suppressing foam activity.

## Conclusions

Aerosolized delivery of *Mycobacterium tuberculosis* necessitates rigorous methodologies and safety considerations. This study demonstrated that silicon antifoaming agent reduces foam formation and retains antifoam efficacy throughout the duration of Mtb aerosol generation procedures. These results also support the conclusion that SAF is a safe and effective foam control agent, exhibiting increased consistency in Mtb aerosol delivery to mice and not impacting bacterial viability.
